# Bone augmentation by octacalcium phosphate and collagen composite coated with poly‐lactic acid cage

**DOI:** 10.1002/cre2.287

**Published:** 2020-03-18

**Authors:** Toshiki Yanagisawa, Ayato Yasuda, Ria I. Makkonen, Shinji Kamakura

**Affiliations:** ^1^ Bone Regenerative Engineering Laboratory, Graduate School of Biomedical Engineering Tohoku University Sendai Japan; ^2^ Faculty of Medicine and Health Technology Tampere University Tampere Finland

**Keywords:** bone regeneration, collagen, octacalcium phosphate, parathyroid hormone, poly‐lactic acid

## Abstract

**Objective:**

Although octacalcium phosphate and collagen composite (OCP/Col) has demonstrated excellent bone regeneration, it has never achieved bone augmentation. The present study investigated whether it could be enabled by OCP/Col disks treated with parathyroid hormone (PTH) and covered with a poly‐lactic acid (PLA) cage.

**Materials and methods:**

The prepared OCP/Col disks with three different types of PLA cages (no hole, one large hole, several small holes) were implanted into subperiosteal pockets in rodent calvaria. Histological, and histomorphometric analyses were conducted at 12 weeks after implantation.

**Results:**

Implants with all PLA cage variants achieved sufficient bone augmentation, and analyses showed that new bone was formed from the original bone and along the PLA cage. While the PLA cage variant with no holes sporadically evoked new bone formation even at the central area of the roof of the PLA cage, the PLA cage variants with holes had no new bone in the area of the hole or beneath the periosteum.

**Conclusions:**

These results suggest that sufficient bone augmentation could be achieved by treating the OCP/Col disks with PTH and covering them with a PLA cage, and periosteum might not have been involved in the bone formation in this experiment.

## 1 INTRODUCTION

After tooth extraction, the residual alveolar ridge generally has limited bone volume because of bone resorption (Tallgren, 2003). Atrophic alveolar ridges might impede proper placement of dental implants in their long‐term functional position, with an acceptable aesthetic profile of the final prosthesis (Buser, Martin, & Belser, 2004). To overcome these challenges, autologous bone grafting has been used for the reconstruction and augmentation of such alveolar ridges to facilitate implantation (Clavero & Lundgren, 2003). However, collecting autologous bones damages the healthy body since there are problems associated with the use of autologous bone in terms of donor site pain, morbidity, and infection (Jensen & Terheyden, 2009). In contrast, hydroxyapatite (HA) and β‐tricalcium phosphate (β‐TCP) have been examined experimentally as alternatives for autologous bone augmentation (Pinholt, Ruyter, Haanaes, & Bang, 1992; Zerbo et al., 2004). Yet, autologous bone grafting is still considered the clinical “gold standard” and the most effective method for bone regeneration (Hjorting‐Hansen, 2002).

Octacalcium phosphate (OCP: Ca_8_H_2_[PO_4_]_6_ 5H_2_O) has been suggested as a precursor of biological apatite in bones (Brown, Mathew, & Tung, 1981; Brown, Smith, Frazier, & Lehr, 1962; Crane, Popescu, Morris, Steenhuis, & Ignelzi Jr., 2006), it enhances bone regeneration more than HA or β‐TCP (Kamakura et al., 2002), and has excellent bioresorbability (Kamakura et al., 1997; Kamakura et al., 1999). Although OCP possesses many desirable properties, its inherent brittleness makes it hard to maintain its shape and precludes its use in clinical applications. Therefore, an OCP and collagen composite (OCP/Col) has been developed to overcome the limitations of OCP in terms of moldability and handling performance (Kamakura, Sasaki, Honda, Anada, & Suzuki, 2006). OCP/Col enhanced bone regeneration more than OCP per se, β‐TCP and collagen composite (β‐TCP/Col) or HA and collagen composite (HA/Col) (Kamakura et al., 2006; Kamakura et al., 2007). Additionally, it promotes osteogenic differentiation and angiogenesis (Kouketsu et al., 2019). After the confirmation of its safety and efficacy in preclinical studies with bone defects (Kawai et al., 2011; Matsui et al., 2014; K. Matsui et al., 2010; Tanuma et al., 2013), a doctor‐initiated and a sponsor‐initiated clinical trials were conducted, (Kawai et al., 2014; Kawai et al., 2016; Kawai et al., 2017; Miura et al., 2019) and the commercialization of OCP/Col in Japan was recently approved for the bone defects in the field of oral surgery.

However, OCP/Col discs alone did not enhance appositional bone formation because of compression stress under the periosteum (Suzuki et al., 2009). Another study has shown strong indications that supporting the OCP/Col discs with a polytetrafluoroethylene (PTFE) ring with higher modulus than OCP/Col alleviates mechanical stress and enables bone formation (A. Matsui et al., 2010). It was also suggested that OCP/Col with the single local administration of teriparatide (TPTD), a crucial regulator of calcium and phosphate metabolism and functions (Sibai, Morgan, & Einhorn, 2011), enhanced bone regeneration in a rodent calvarial critical‐sized bone defect more than OCP/Col per se (Kajii et al., 2018).

Therefore, it was hypothesized that bone augmentation could be achieved by covering the parathyroid hormone (PTH) treated OCP/Col discs with poly‐lactic acid (PLA) cages. To study this hypothesis, these samples were implanted into subperiosteal pockets of rodent calvarium. Additionally, it was also investigated if bone regeneration was influenced by the condition of contact between the periosteum and OCP/Col. Previous studies have indicated that bone augmentation instigated by OCP/Col is predominantly initiated from the surface of original bone side and no bone formation has been observed from the periosteal side (A. Matsui, Anada, et al., 2010; Suzuki et al., 2009), even though it has been suggested that periosteal cells formed bone (Nakahara et al., 1990).

## 2 MATERIALS AND METHODS

### 2.1 Preparation of OCP/Col

The preparation of octacalcium phosphate and collagen composite (OCP/Col) was described previously (Kajii et al., 2018). Briefly, OCP was prepared by direct precipitation, and sieved granules (particle sizes 300–500 μm) were produced. Collagen was prepared from NMP collagen PS (Nippon Meat Packers, Tsukuba, Ibaraki, Japan), which is a lyophilized powder of pepsin‐digested atelocollagen isolated from porcine dermis. Sieved granules of OCP were added to concentrated collagen and mixed, and the weight percentage of OCP in OCP/Col was 77%. The OCP/Col mixture was then lyophilized, and a disk was molded (diameter 9 mm, thickness 1.5 mm). OCP/Col disks were prepared by dehydrothermal treatment (150°C, 24 hr) in a vacuum drying oven. OCP/Col disks were sterilized using electron beam irradiation.

### 2.2 Preparation of PLA cages

**Figure 1:**
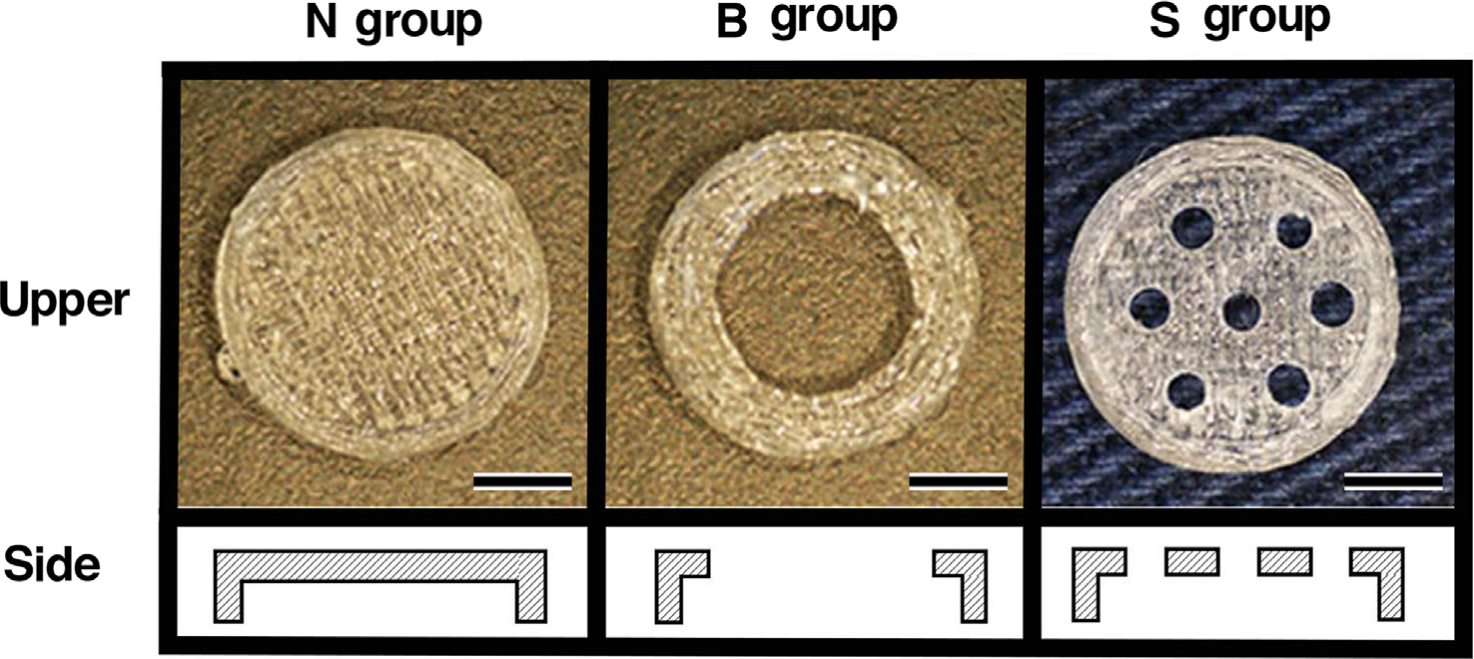
Poly‐lactic acid (PLA) cages of the N, B, and S groups. The PLA cage was 10 mm outside diameter, 8.5 mm inside diameter, 2.5 mm outside height, and 1.5 mm inside height. The cage covered the upper surface and side of the octacalcium phosphate and collagen composite (OCP/Col) disc. The PLA cages had three variants (no hole (N), one large hole (B): φ6 mm × 1, and several small holes (S): φ1 mm × 7), Bars: 3 mm

PLA cages were prepared using a 3D printer (Makerbot replicator desktop 3D printer, Makerbot Industries, NY). The PLA cages were formed as follows: The design file of the cage was converted into a data file used by the exclusive software of the 3D printer and transferred to the 3D printer. A cage was formed using the 3D printer with melted PLA (Makerbot PLA Filament, Makerbot Industries) filament, and with a 10 mm outside diameter, 8.5 mm inside diameter, 2.5 mm outside height, and 1.5 mm inside height. While the cage covered the upper surface and the sides of the OCP/Col disc, there was no cover for the lower surface of OCP/Col disc. The PLA cages had three variants of upper side (no hole (N), one large hole (B): φ6 mm × 1, and several small holes (S): φ1 mm × 7) (Figure 1).

### 2.3 Preparation of TPTD solution

Chemically synthesized TPTD, an active ingredient of Teribone Inj. 56.5 μg (Asahi Kasei Pharma Corp., Tokyo, Japan), was used. Lyophilized TPTD acetate was reconstituted with saline at a concentration of 50 μg/ml. The final TPTD solution was prepared by diluting TPTD solution (1.0 μg/0.1 ml) with saline. The prepared TPTD solution (1.0 μg/0.1 ml) was stored in a freezer (−20°C) until immediately, before use.

### 2.4 Implantation procedure

**Figure 2:**
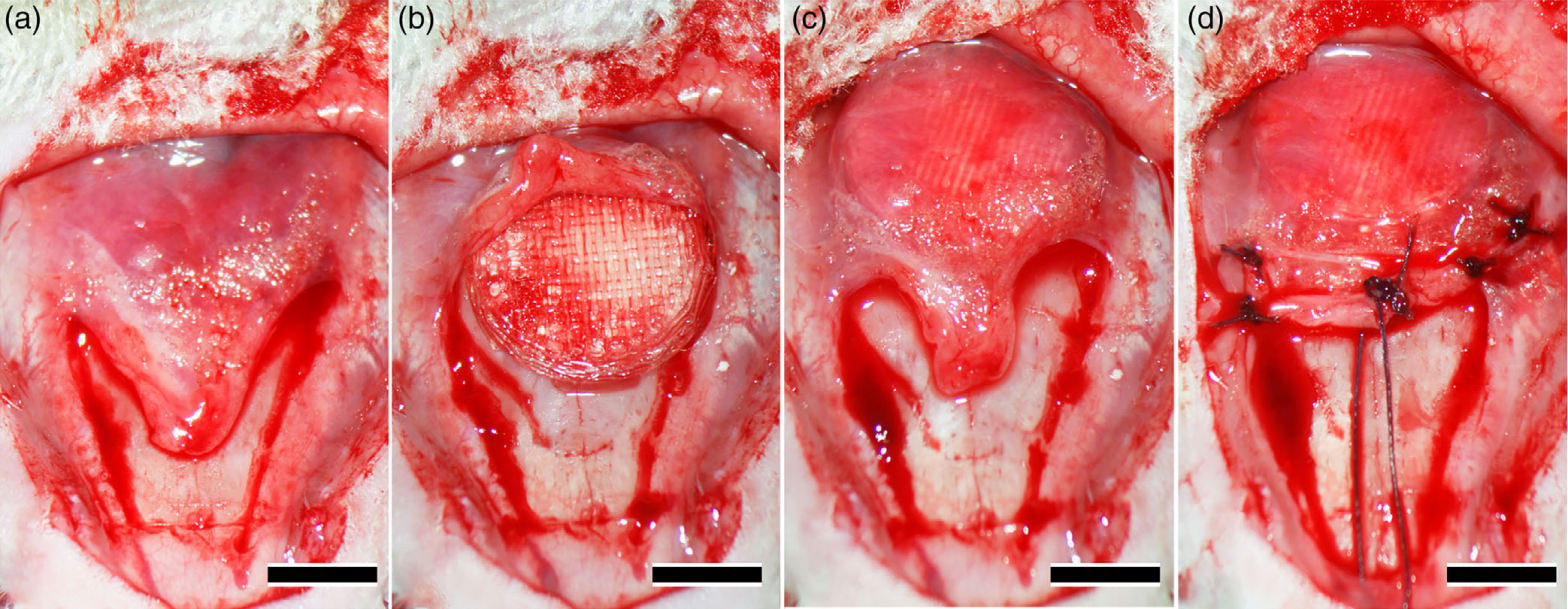
Implantation procedure of samples. The periosteum of the calvarium was ablated after the incision reached the skull, and a subperiosteal pocket was prepared on the calvarium (a). An octacalcium phosphate and collagen composite (OCP/Col) disk treated with parathyroid hormone (PTH) (1.0 μg/0.1 ml) was covered with a poly‐lactic acid (PLA) cage (sample), and it was placed on the calvarium (b). The sample was inserted into the created subperiosteal pocket by moving it backward on the bone surface and placed under the periosteum so that it was in contact with the bone surface of the calvarium (c). After that the sample was covered with the ablated periosteum and the incision was sutured with absorbable thread to prevent the sample from escaping the subperiosteal pocket (d). Bars: 3 mm

Twelve‐week‐old male Wistar rats (SLC Corp., Hamamatsu, Shizuoka, Japan) were used. The principles of laboratory animal care as well as national laws were followed. All procedures were approved by the Animal Research Committee of Tohoku University (2016BeA‐001). Experimental rats were anesthetized with intraperitoneal dexmedetomidine hydrochloride (0.05 mg/kg), midazolam (0.12 mg/kg), and butorphanol tartrate (0.15 mg/kg). First, an accurate skin incision was made on the forehead region and the dissected skin was ablated. Then, periosteum of the calvarium was ablated after the incision reached the skull, and a subperiosteal pocket was prepared on the calvarium (Figure 2a). An OCP/Col disk treated with PTH (1.0 μg/0.1 ml) was covered with a PLA cage (sample), and it was placed on the calvarium (Figure 2b). The sample was inserted into the created subperiosteal pocket by moving it backward on the calvarium and placed under the periosteum so that it was in contact with the bone surface of the calvarium (Figure 2c). After that the sample was covered with the ablated periosteum and the incision was sutured with absorbable thread, to prevent the sample from escaping the subperiosteal pocket (Figure 2d). Finally, the dissected skin was repositioned and sutured with silk thread. Each five experimental rats per group were randomly divided into three groups (N, B, and S groups), and in rats in each group were fixed 12 weeks after implantation (*n* = 5 × 3).

### 2.5 Micro‐CT examination

4 and 12 weeks after implantation, in vivo microcomputed tomography (CT) analysis of the rat calvarium was performed using an X‐ray CT system (Latheta LCT‐200; Hitachi Aloka Medical, Tokyo, Japan) after intraperitoneal injection of sodium pentobarbital (50 mg/kg), as described previously (Kajii et al., 2018). The calvariums were scanned continuously in increments of 120 μm, with pixel size 60 μm. CT images were acquired using the following parameters: 50 kVp tube voltage, 500 μA tube current. After completion of the CT analysis at 12 weeks, rats were euthanized by an intraperitoneal injection of an overdose of sodium pentobarbital. After sacrifice, the implants were resected together with the surrounding bones and tissues and fixed in 4% paraformaldehyde in 0.1 M phosphate‐buffered saline, pH 7.4.

### 2.6 Tissue preparations and quantitative micrograph analysis

**Figure 3:**
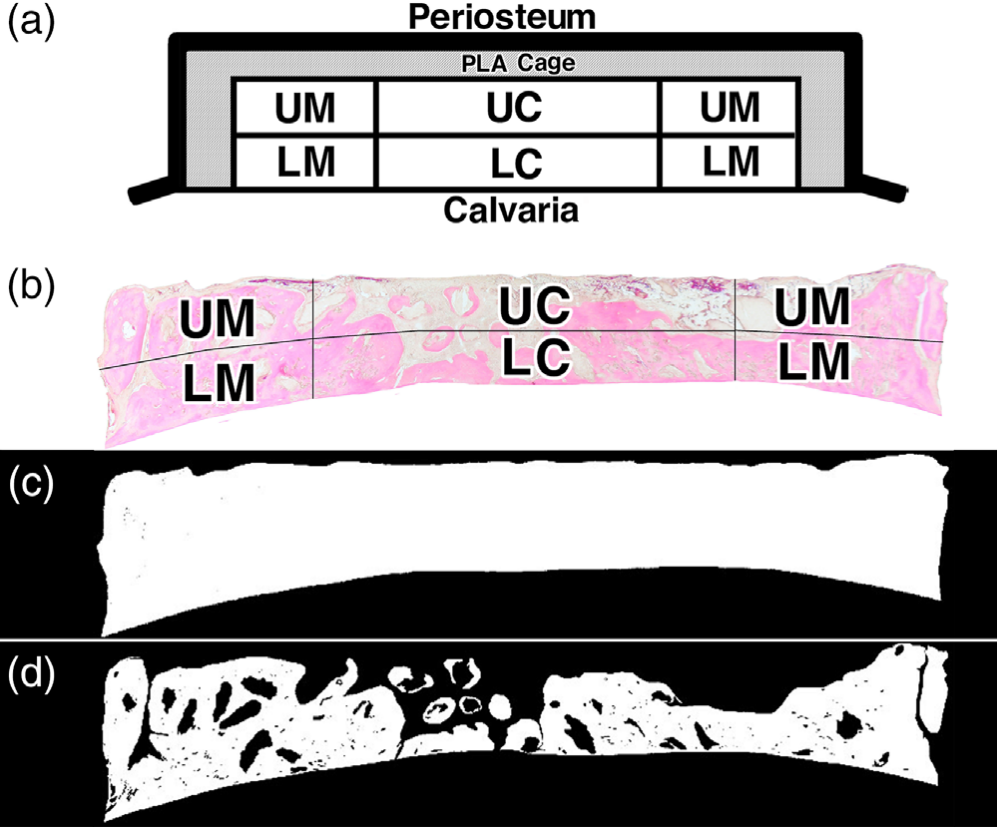
Histomorphometrically analysis of histological sections. Histomorphometrical analysis of the histological sections of the implanted sample discs (a) divided into four areas (b) (lower marginal [LM], upper marginal [UM], lower central [LC], and upper central [UC]). The percentage of newly formed bone (n‐Bone%) was calculated as the area of newly formed bone/the area surrounded by the cage (c, d) × 100 for all areas and the area in each region

The samples were decalcified in 10% ethylenediaminetetraacetic acid in 0.01 M phosphate buffer, pH 7.4 at 4°C for 4–6 weeks after radiographs had been taken. Specimens were embedded in paraffin, and the center was extracted, and sectioned coronally. Sections were stained with hematoxylin and eosin, and photographs were taken using a photomicroscope (Leica DM2500, Leica Microsystems Japan, Tokyo, Japan). Histomorphometrical analysis of the histological sections of implanted OCP/Col (Figure 3a) involved dividing the sections into four areas (lower marginal [LM], upper marginal [UM], lower central [LC], and upper central [UC]) (Figure 3b). The percentage of newly formed bone (n‐Bone%) was calculated as the area of newly formed bone/the area surrounded by the cage × 100 for all areas and the area of each region (Figure 3c, d). n‐Bone% was quantified two‐dimensionally using ImageJ version 1.43 (National Institutes of Health, Bethesda, MD) public domain software.

### 2.7 Statistical analysis

At 12 weeks after implantation, n‐Bone% was analyzed statistically using Excel v. X. (Microsoft Co., Redmond, WA). All values are reported as mean ± *SD*. The *χ* ^2^ test was used to investigate whether each group had a normal distribution, and Bartlett's test was employed to examine the homogeneity of variance across samples. One‐way analysis of variance or the Kruskal–Wallis test was used to compare means among groups. Significance was accepted at *p* < .05. If significant differences were detected in the mean values, Tukey–Kramer or Scheffe's multiple comparison analysis was used as a post hoc test.

## 3 RESULTS

### 3.1 Micro‐CT analysis

**Figure 4:**
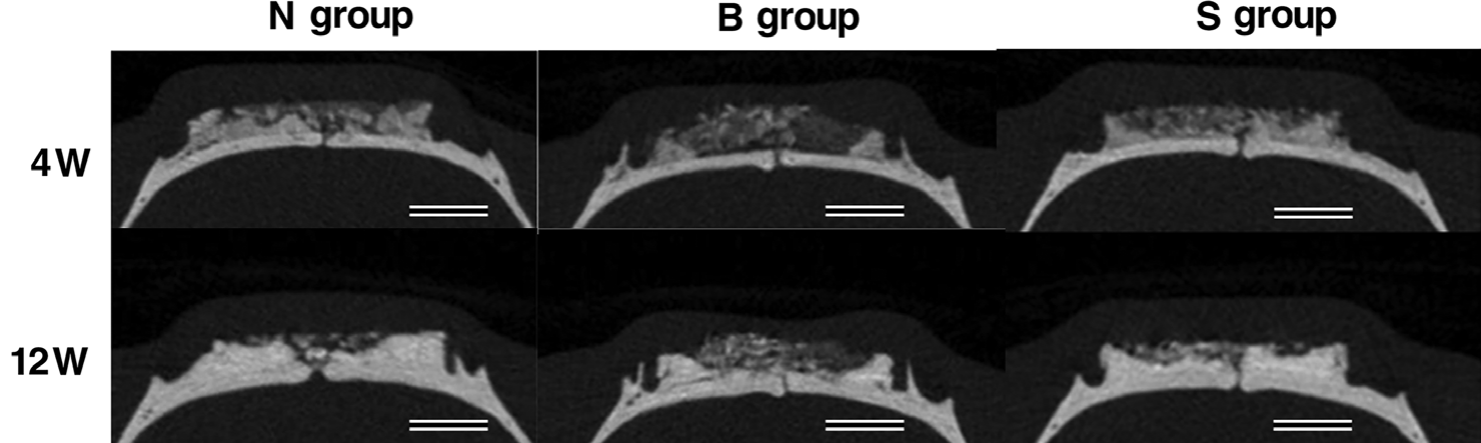
Micro‐computed tomography (CT) at 4 and 12 weeks after implantation. The top of the images indicate the skin side, and the bottom is the original bone side. In the N and S groups, radiopacity in the octacalcium phosphate and collagen composite (OCP/Col) implanted area was more abundant than radiolucency in the area at 4 weeks after implantation. After 12 weeks, the increased radiopacity in marginal area was greater than that in central area. In the B group, radiolucency in the OCP/Col implanted area was more abundant than radiopacity in the area at 4 weeks after implantation. After 12 weeks, the radiopacity had increased in the OCP/Col implanted area, but it was still lower than other groups. Bars: 3 mm

The top side of the images indicate the skin side, whereas the bottom side shows the original bone side. In the N and S groups, radiopacity in the OCP/Col implanted area was more abundant than radiolucency in the area at 4 weeks after implantation. The radiopacity was dominant in marginal areas and near the original bone and increased with time. After 12 weeks, the increased radiopacity was greater in the marginal area than in the central area. In the B group, radiolucency in the OCP/Col implanted area was more abundant than radiopacity in the area at 4 weeks after implantation. Although the radiopacity was dominant in marginal areas and increased with time, it was lower than the radiopacity in other groups. After 12 weeks, the radiopacity had increased in the OCP/Col implanted area, but was still lower than in the other groups (Figure 4).

### 3.2 Histological results of implants

**Figure 5:**
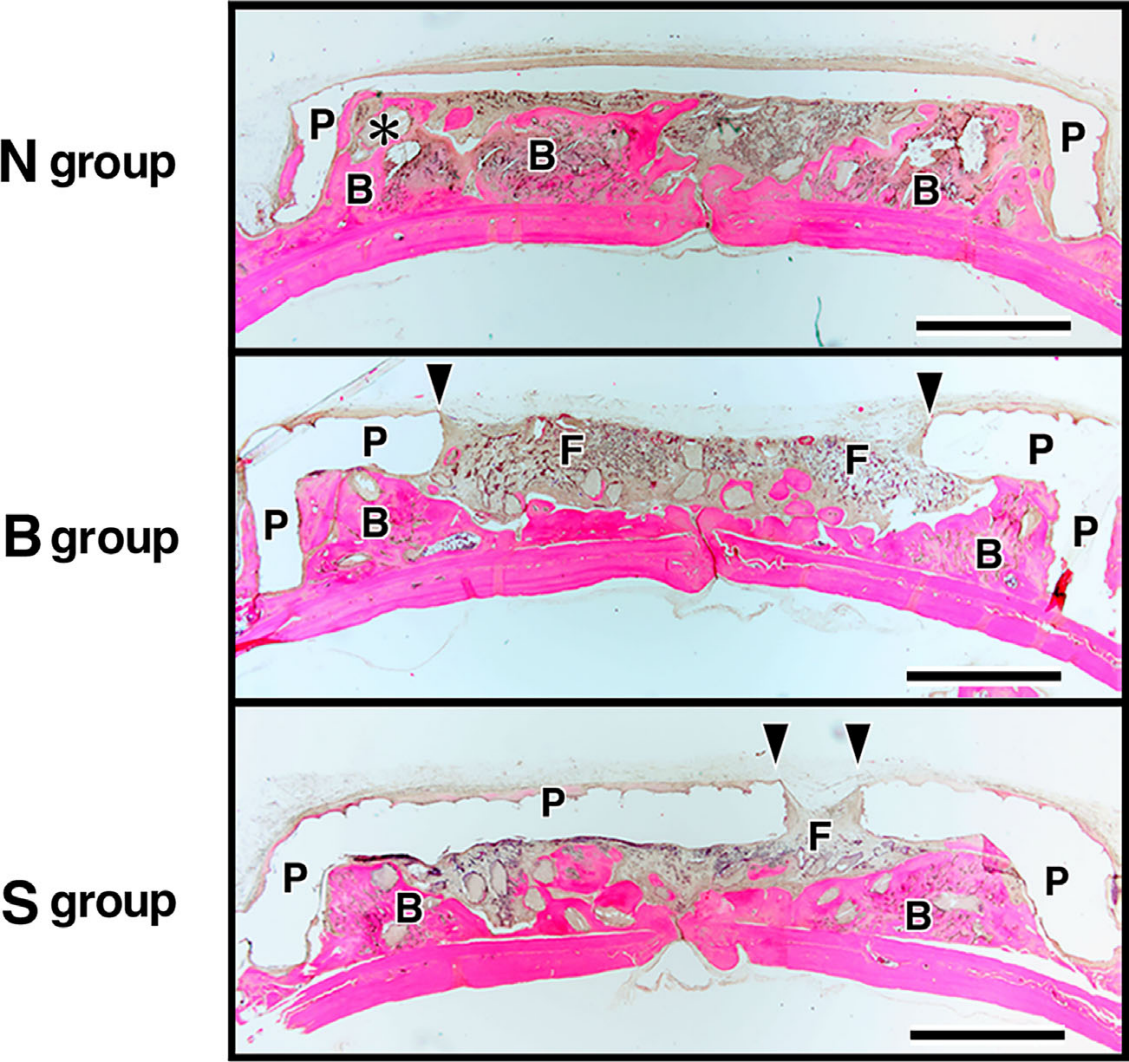
Histological results at 12 weeks after implantation for the total area of experimental group. The top of the figure indicates the skin side, and the bottom is the original bone side. It was observed bone augmentation in all groups (N, B, and S), and newly formed bone was extended from the original bone toward the skin side and developed along the poly‐lactic acid (PLA) cage. Bars: 3 mm; B, newly formed bone; P, PLA cage; F, fibrous tissue

**Figure 6:**
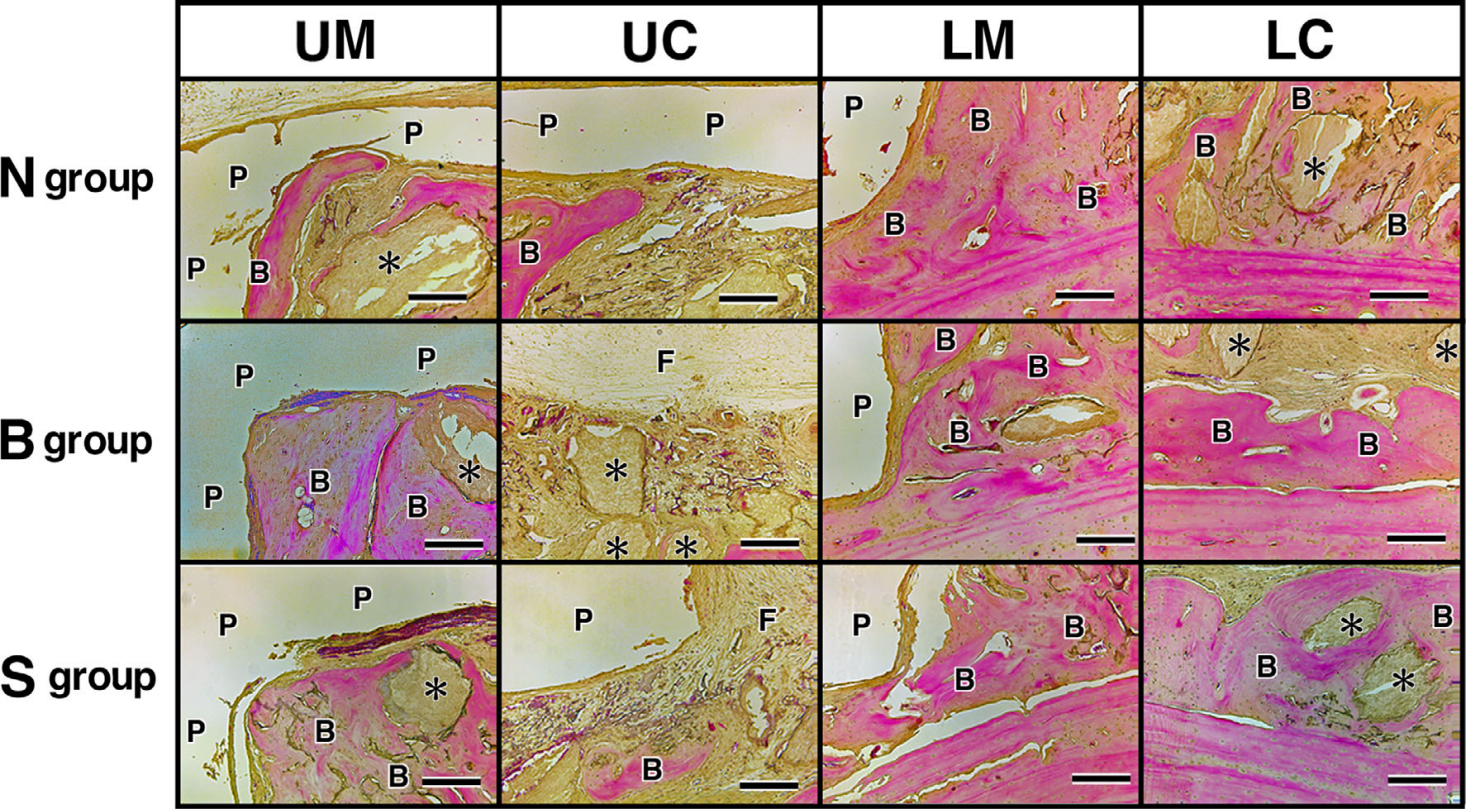
Histological results at 12 weeks after implantation in each section by experimental group. The top of the figure indicates the skin side, and the bottom is the original bone side. Each section was composed of upper marginal (UM), upper central (UC), lower marginal (LM), and lower central (LC). Newly formed bone was nucleated by the implanted octacalcium phosphate and collagen composite (OCP/Col) and extended from the original bone toward the skin side and developed along the poly‐lactic acid (PLA) cage. And it occasionally, contacted with the roof of the PLA cage (N‐UM and UC, B‐UM, and S‐UM). It was abundant near the original bone and in the marginal area, and it demonstrated a mosaic pattern (N‐LM and LC, B‐LM and LC, and S‐LM and LC). In the N group, the newly formed bone was contacted with the roof of the PLA cage including central area. (N‐UM and UC). In the PLA holed groups (B and S groups), fibrous tissue filled the hole in the PLA cage and the area near the hole, and there was no bone in the hole of the PLA cage and beneath the periosteum (B‐UC and S‐UC). However, newly formed bone was occasionally observed on the skin side covered by the PLA cage in the S group (S‐UC). Bars: 200 μm; asterisk, OCP/Col; B, newly formed bone; P, PLA cage; F, fibrous tissue

The top side of the images indicate the skin side, whereas the bottom side shows the original bone side. Bone augmentation was observed in all groups (N, B, and S groups), where the implanted discs of OCP/Col were treated with PTH and covered with a PLA cage. Newly formed bone was nucleated by the implanted OCP/Col and extended from the original bone toward the skin side and along the PLA cage and was occasionally in contact with the roof of the PLA cage. New bone was abundant near the original bone and the marginal area, and it demonstrated a mosaic pattern. In the N group, the newly formed bone was sporadically in contact with the roof of the PLA cage even in the central area. In the PLA holed groups (B and S groups), fibrous tissue filled the holes in the PLA cage and the area near the hole, and no bone formation could be observed in the hole of the PLA cage or beneath the periosteum. However, in some samples with S type PLA cages, newly formed bone was observed on the skin side of the covered sample (Figures 5 and 6).

### 3.3 Histomorphometrical examination

**Figure 7:**
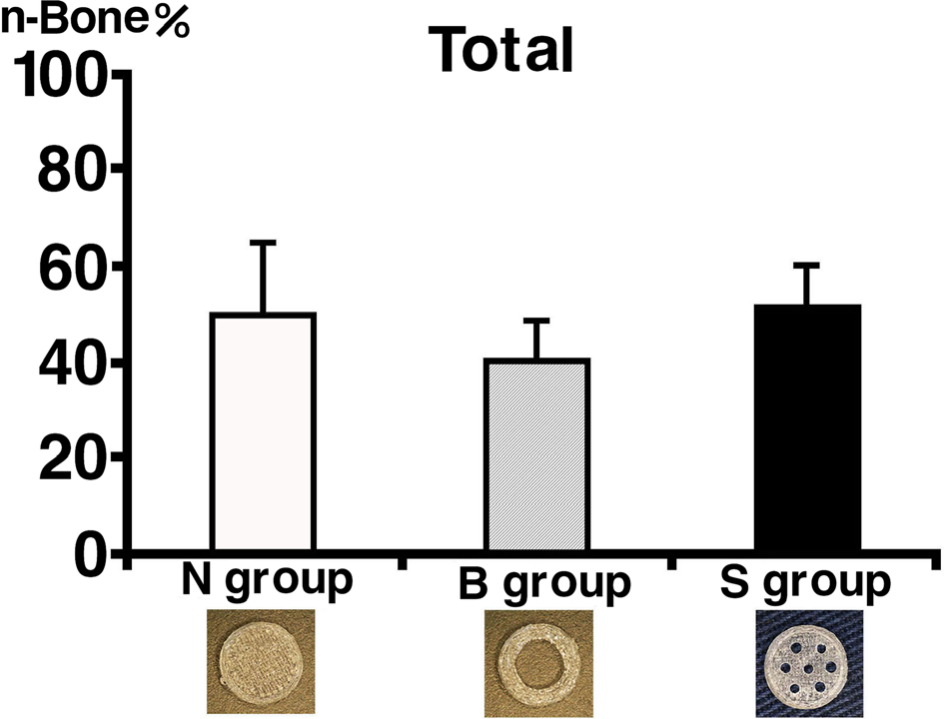
Quantitative analysis of newly formed bone of total area of experimental group. Although n‐Bone% of S group in total area was higher than those of N group and B group, there was no significant difference among these groups

**Figure 8:**
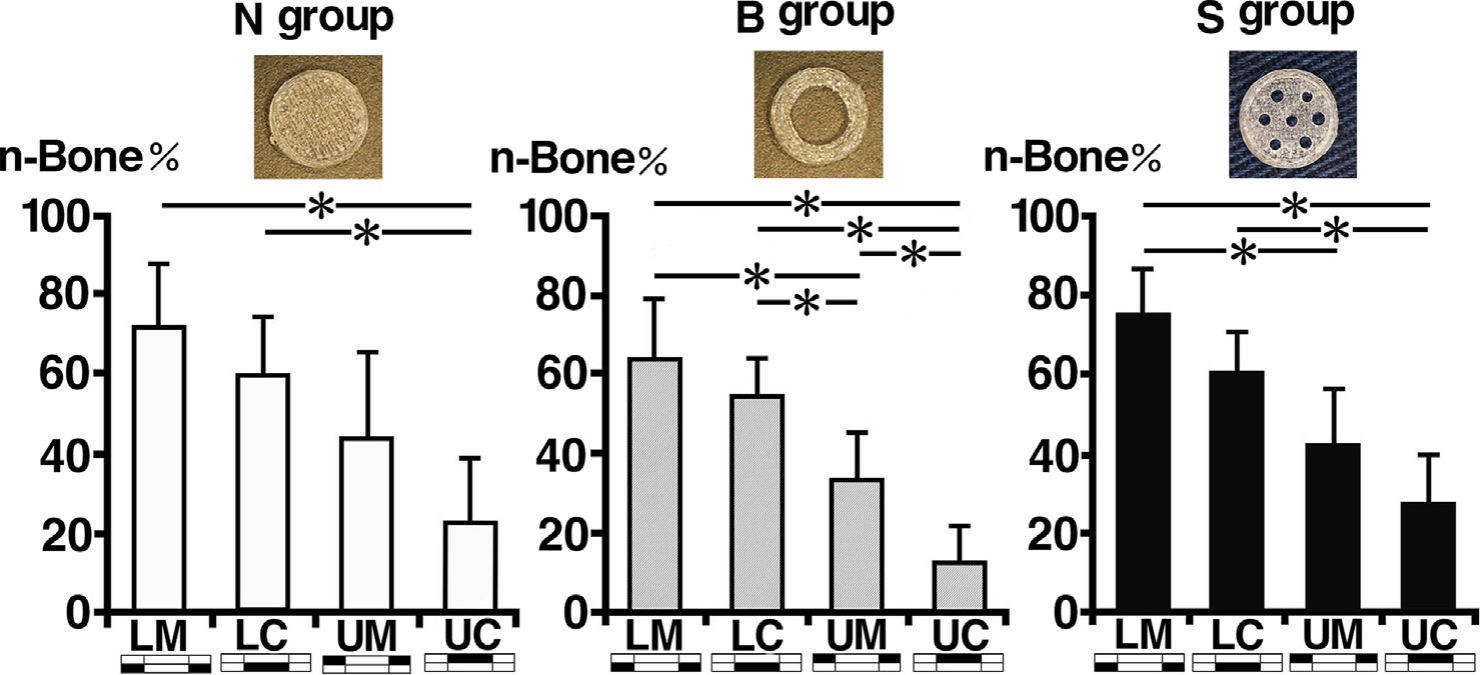
Quantitative analysis of newly formed bone in each section by experimental group. In every group, the percentage of newly formed bone (n‐Bone%) in the lower marginal (LM) area was highest, followed in order by lower central (LC), upper marginal (UM), and upper central (UC). In the N group, the n‐Bone% of UC was significantly lower than those of LM and LC. In the B group, the n‐Bone% of UC was significantly lower than those of LM, LC, and UM. Additionally, the n‐Bone% of UM was significantly lower than those of LM and LC. In the S group, the n‐Bone% of UC was significantly lower than those of LM and LC, and the n‐Bone% of UM was significantly lower than that of LM

The n‐Bone% of the total area in each section (LM, LC, UM, and UC) of the experimental groups is shown in Figures 7 and 8 and Table 1. Although the n‐Bone% in total area of the S group (52.0 ± 7.4%) was higher than those of the N group (50.3 ± 13.4%) and B group (41.0 ± 7.3%), there was no significant difference among these groups (Figure 7). In every group, the n‐Bone% of LM was highest, followed in order by LC, UM, and UC. In the N group, the n‐Bone% of UC (23.0 ± 15.2%) was significantly lower than those of LM (72.5 ± 14.2%) and LC (60.5 ± 13.2%). In the B group, the n‐Bone% of UC (13.4 ± 7.5%) was significantly lower than those of LM (64.6 ± 13.4%), LC (55.4 ± 8.5%), and UM (34.0 ± 10.7%). In addition, the n‐Bone% of UM (34.0 ± 10.7%) was significantly lower than those of LM (64.6 ± 13.4%) and LC (55.4 ± 8.5%). In the S group, the n‐Bone% of UC (27.6 ± 11.6%) was significantly lower than those of LM (75.3 ± 10.4%) and LC (61.2 ± 9.1%), and the n‐Bone% in the S group of UM (42.7 ± 13.0%) was significantly lower than that of LM (75.3 ± 10.4%) (Figure 8).

**Table 1:** Quantitative analysis of the percentage of newly formed bone (n‐Bone%) for the total area and in each section by experimental group

Area	N group	B group	S group
Total	50.3 ± 13.4%	41.0 ± 7.3%	52.0 ± 7.4%
LM	72.5 ± 14.2%	64.6 ± 13.4%	75.3 ± 10.4%
LC	60.5 ± 13.2%	55.4 ± 8.5%	61.2 ± 9.1%
UM	44.9 ± 19.1%	34.0 ± 10.7%	42.7 ± 13.0%
UC	23.0 ± 15.2%	13.4 ± 7.5%	27.6 ± 11.6%

Abbreviations: LC, lower central; LM, lower marginal; UC, upper central; UM, upper marginal.

## 4 DISCUSSION

This study confirmed bone augmentation of nearly double the thickness of original bone, when PTH treated and PLA covered OCP/Col discs were implanted into the subperiosteal pocket on a rodent calvarium. Although this study provided different bone augmentation models, it surpassed the results of previous studies of bone augmentation using OCP/Col (A. Matsui, Anada, et al., 2010; Suzuki et al., 2009). It was reported that single local administration of TPTD with OCP/Col promoted bone regeneration in rodent calvarial defects more than OCP/Col without TPTD (Kajii et al., 2018). Administration of TPTD is expected to accelerate remodeling of newly formed bone by enhancing osteogenic and osteoclastic activities (Morimoto et al., 2014), and thus adding TPTD to OCP/Col is hypothesized to promote bone augmentation. Additionally, PLA cage preserved the shape of OCP/Col disc and inhibited invasion of fibrous tissue into the implants. When the upper side of OCP/Col was covered by PLA cage, the newly formed bone was occasionally in contact with the roof of the PLA cage including the central area. However, this was never observed when OCP/Col was covered by only lateral walls (A. Matsui, Anada, et al., 2010). Similarly, the PLA cage with holes on the top had no bone formation along the roof of the implant where fibrous tissue had invaded the OCP/Col. This indicates the possibility that fibrous tissue might inhibit bone formation and that covering OCP/Col completely might promote bone augmentation.

In all experimental groups, the radiopaque area increased with time on the original bone side and in the marginal area from 4 to 12 weeks after implantation. In the central area of the skin side, radiolucency was dominant in B group, whereas radiopaque and radiolucent areas were mixed in the N and S group at 12 weeks after implantation. However, it was reported that it would be more suitable to apply histomorphometric analysis of newly formed bone than radiomorphometric analysis by micro‐CT images (Kajii et al., 2018) since it is difficult to distinguish newly formed bone and converted apatite by radiographic examination. Therefore, histomorphometric analysis was prioritized in this study. In all the experimental groups, the newly formed bone extended from the original bone toward the skin side, and was abundant on the original bone side and in the marginal area. It has previously been shown that OCP acts together with osteoblasts, bone lining cells and their closely committed progenitors on the original bone to enhance bone formation from the surface of the original bone (Sasano et al., 1999). Thus, it can be assumed that in this study osteoprogenitors might have interacted with OCP/Col and consequently enhanced bone augmentation. Additionally, using a PTFE ring to cover the OCP/Col, has been shown to enhance appositional bone formation by alleviating mechanical stress on OCP/Col (A. Matsui, Anada, et al., 2010). Since some of the new bone formation inside the PLA cage occurred in contact with the biocompatible PLA, it is likely that bone formation was initiated by osteoprogenitors that had invaded into the OCP/Col from the blood stream (Miettinen, Makela, Vainio, Rokkanen, & Tormala, 1992).

There were no significant differences of n‐Bone% in the total area among N, B, and S groups. It suggests that administration of TPTD or covering by a PLA cage contribute the bone augmentation with OCP/Col more than the hole variants at the top of the PLA cages. In contrast, the n‐Bone% of LM (64.6–75.3%) was highest, followed in order by those of LC (55.4–61.2%), UM (34.0–44.9%), and UC (13.4–27.6%) in all experimental groups. The n‐Bone% of upper central area (UC) in the S group (27.6%) was highest, followed in order by the N group (23.0%) and the B group (13.4%). Because UC of the B group was not covered by the PLA cage and was directly in contact with periosteum, the extensive fibrous tissue invasion might have inhibited new bone formation (A. Matsui, Anada, et al., 2010). In contrast, fibrous tissue invasion from the small holes in the S group may have had partial influence on delaying new bone formation. Although it was previously reported that the periosteum was involved in new bone formation (Nakahara et al., 1990), no bone had formed near the hole area of the PLA cage. This suggests that, in case of this experiment, periosteum might not have been involved in bone formation. The n‐Bone% of upper marginal area (UM) in the N group (44.9%) was highest, followed in order by the S group (42.7%) and the B group (34.0%). Because this area was partially covered by PLA, the influence of invaded fibrous tissue might have been diminished. The n‐Bone% of lower central area (LC) in the S group (61.2%) was highest, followed in order by the N group (60.5%) and B group (55.4%). And The n‐Bone% of lower marginal area (LM) was highest in the S group (75.3%), followed in order by the N group (72.5%) and B group (64.6%). This suggests that bone augmentation in this area would be mainly initiated by osteoprogenitors which invaded into OCP/Col and migrated along the PLA cage.

Although this study demonstrated sufficient bone augmentation initiated by OCP/Col treated with PTH and covered with a PLA cage, PLA remains in the body for a long time. Additionally, the bone augmentation using this conventional OCP/Col (OCP/Col in this study) was insufficient because of the poor mechanical properties of this material (A. Matsui, Anada, et al., 2010). To achieve bone augmentation using OCP/Col itself, a new OCP/Col should be developed to maintain the shape and improve the mechanical properties after implantation.

## CONFLICT OF INTEREST

One of the authors (S.K.) has obtained patents on OCP/Col in Japan (#5046511) and combination of calcium phosphate containing porous composite and PTH in Japan (#6094716).

## References

[cre2287-bib-0001] Brown, W. E. , Mathew, M. , & Tung, M. S. (1981). Crystal‐chemistry of octacalcium phosphate. Progress in Crystal Growth and Characterization of Materials, 4(1–2), 59–87. 10.1016/0146-3535(81)90048-4

[cre2287-bib-0002] Brown, W. E. , Smith, J. P. , Frazier, A. W. , & Lehr, J. R. (1962). Crystallographic and chemical relations between octacalcium phosphate and hydroxyapatite. Nature, 196(4859), 1050–1055. 10.1038/1961050a0

[cre2287-bib-0003] Buser, D. , Martin, W. , & Belser, U. C. (2004). Optimizing esthetics for implant restorations in the anterior maxilla: Anatomic and surgical considerations. The International Journal of Oral & Maxillofacial Implants, 19(Suppl), 43–61. 15635945

[cre2287-bib-0004] Clavero, J. , & Lundgren, S. (2003). Ramus or chin grafts for maxillary sinus inlay and local onlay augmentation: Comparison of donor site morbidity and complications. Clinical Implant Dentistry and Related Research, 5(3), 154–160. 10.1111/j.1708-8208.2003.tb00197.x 14575631

[cre2287-bib-0005] Crane, N. J. , Popescu, V. , Morris, M. D. , Steenhuis, P. , & Ignelzi, M. A., Jr. (2006). Raman spectroscopic evidence for octacalcium phosphate and other transient mineral species deposited during intramembranous mineralization. Bone, 39(3), 434–442. 10.1016/j.bone.2006.02.059 16627026

[cre2287-bib-0006] Hjorting‐Hansen, E. (2002). Bone grafting to the jaws with special reference to reconstructive preprosthetic surgery. A historical review. Mund‐, Kiefer‐ Und Gesichtschirurgie, 6(1), 6–14. 10.1007/s10006-001-0343-6 11974547

[cre2287-bib-0007] Jensen, S. S. , & Terheyden, H. (2009). Bone augmentation procedures in localized defects in the alveolar ridge: Clinical results with different bone grafts and bone‐substitute materials. The International Journal of Oral & Maxillofacial Implants, 24(Suppl), 218–236. 19885447

[cre2287-bib-0008] Kajii, F. , Iwai, A. , Tanaka, H. , Matsui, K. , Kawai, T. , & Kamakura, S. (2018). Single‐dose local administration of teriparatide with a octacalcium phosphate collagen composite enhances bone regeneration in a rodent critical‐sized calvarial defect. Journal of Biomedical Materials Research. Part B, Applied Biomaterials, 106(5), 1851–1857. 10.1002/jbm.b.33993 28922546 PMC6032915

[cre2287-bib-0009] Kamakura, S. , Sasaki, K. , Homma, T. , Honda, Y. , Anada, T. , Echigo, S. , & Suzuki, O. (2007). The primacy of octacalcium phosphate collagen composites in bone regeneration. Journal of Biomedical Materials Research. Part A, 83(3), 725–733. 10.1002/jbm.a.31332 17559110

[cre2287-bib-0010] Kamakura, S. , Sasaki, K. , Honda, Y. , Anada, T. , & Suzuki, O. (2006). Octacalcium phosphate combined with collagen orthotopically enhances bone regeneration. Journal of Biomedical Materials Research. Part B, Applied Biomaterials, 79(2), 210–217. 10.1002/jbm.b.30531 16615073

[cre2287-bib-0011] Kamakura, S. , Sasano, Y. , Homma, H. , Suzuki, O. , Kagayama, M. , & Motegi, K. (1999). Implantation of octacalcium phosphate (OCP) in rat skull defects enhances bone repair. Journal of Dental Research, 78(11), 1682–1687. 10.1177/00220345990780110401 10576163

[cre2287-bib-0012] Kamakura, S. , Sasano, Y. , Homma‐Ohki, H. , Nakamura, M. , Suzuki, O. , Kagayama, M. , & Motegi, K. (1997). Multinucleated giant cells recruited by implantation of octacalcium phosphate (OCP) in rat bone marrow share ultrastructural characteristics with osteoclasts. Journal of Electron Microscopy, 46(5), 397–403. 10.1093/oxfordjournals.jmicro.a023535 9394452

[cre2287-bib-0013] Kamakura, S. , Sasano, Y. , Shimizu, T. , Hatori, K. , Suzuki, O. , Kagayama, M. , & Motegi, K. (2002). Implanted octacalcium phosphate is more resorbable than beta‐tricalcium phosphate and hydroxyapatite. Journal of Biomedical Materials Research, 59(1), 29–34. 10.1002/jbm.1213 11745534

[cre2287-bib-0014] Kawai, T. , Echigo, S. , Matsui, K. , Tanuma, Y. , Takahashi, T. , Suzuki, O. , & Kamakura, S. (2014). First clinical application of octacalcium phosphate collagen composite in human bone defect. Tissue Engineering. Part A, 20(7–8), 1336–1341. 10.1089/ten.TEA.2013.0508 24294829 PMC3993018

[cre2287-bib-0015] Kawai, T. , Matsui, K. , Iibuchi, S. , Anada, T. , Honda, Y. , Sasaki, K. , … Echigo, S. (2011). Reconstruction of critical‐sized bone defect in dog skull by octacalcium phosphate combined with collagen. Clinical Implant Dentistry and Related Research, 13(2), 112–123. 10.1111/j.1708-8208.2009.00192.x 19438952

[cre2287-bib-0016] Kawai, T. , Suzuki, O. , Matsui, K. , Tanuma, Y. , Takahashi, T. , & Kamakura, S. (2017). Octacalcium phosphate collagen composite facilitates bone regeneration of large mandibular bone defect in humans. Journal of Tissue Engineering and Regenerative Medicine, 11(5), 1641–1647. 10.1002/term.2110 26612731

[cre2287-bib-0017] Kawai, T. , Tanuma, Y. , Matsui, K. , Suzuki, O. , Takahashi, T. , & Kamakura, S. (2016). Clinical safety and efficacy of implantation of octacalcium phosphate collagen composites in tooth extraction sockets and cyst holes. Journal of Tissue Engineering, 7, 2041731416670770. 10.1177/2041731416670770 PMC5051665 27757220

[cre2287-bib-0018] Kouketsu, A. , Matsui, K. , Kawai, T. , Ezoe, Y. , Yanagisawa, T. , Yasuda, A. , … Kamakura, S. (2019). Octacalcium phosphate collagen composite stimulates the expression and activity of osteogenic factors to promote bone regeneration. Journal of Tissue Engineering and Regenerative Medicine, 14, 99–107. 10.1002/term.2969 31721475 PMC7027853

[cre2287-bib-0019] Matsui, A. , Anada, T. , Masuda, T. , Honda, Y. , Miyatake, N. , Kawai, T. , … Suzuki, O. (2010). Mechanical stress‐related calvaria bone augmentation by onlayed octacalcium phosphate‐collagen implant. Tissue Engineering. Part A, 16(1), 139–151. 10.1089/ten.TEA.2009.0284 19642866

[cre2287-bib-0020] Matsui, A. , Matsui, K. , Handa, T. , Tanuma, Y. , Miura, K. , Kato, Y. , … Echigo, S. (2014). The regenerated bone quality by implantation of octacalcium phosphate collagen composites in a canine alveolar cleft model. The Cleft Palate‐Craniofacial Journal, 51(4), 420–430. 10.1597/12-096 23369014

[cre2287-bib-0021] Matsui, K. , Matsui, A. , Handa, T. , Kawai, T. , Suzuki, O. , Kamakura, S. , & Echigo, S. (2010). Bone regeneration by octacalcium phosphate collagen composites in a dog alveolar cleft model. International Journal of Oral and Maxillofacial Surgery, 39(12), 1218–1225. 10.1016/j.ijom.2010.07.015 20863660

[cre2287-bib-0022] Miettinen, H. , Makela, E. A. , Vainio, J. , Rokkanen, P. , & Tormala, P. (1992). The effect of an intramedullary biodegradable self‐reinforced polyglycolic acid implant on tubular bone. An experimental study on growing dogs. Journal of Biomaterials Science. Polymer Edition, 3(6), 435–442. 10.1163/156856292X00411 1329935

[cre2287-bib-0023] Miura, K. I. , Sumita, Y. , Kajii, F. , Tanaka, H. , Kamakura, S. , & Asahina, I. (2019). First clinical application of octacalcium phosphate collagen composite on bone regeneration in maxillary sinus floor augmentation: A prospective, single‐arm, open‐label clinical trial. Journal of Biomedical Materials Research. Part B, Applied Biomaterials, 108, 243–252. 10.1002/jbm.b.34384 30980703

[cre2287-bib-0024] Morimoto, T. , Kaito, T. , Kashii, M. , Matsuo, Y. , Sugiura, T. , Iwasaki, M. , & Yoshikawa, H. (2014). Effect of intermittent administration of teriparatide (parathyroid hormone 1‐34) on bone morphogenetic protein‐induced bone formation in a rat model of spinal fusion. The Journal of Bone and Joint Surgery. American Volume, 96(13), e107–e1‐8. 10.2106/jbjs.M.01097 24990981

[cre2287-bib-0025] Nakahara, H. , Bruder, S. P. , Haynesworth, S. E. , Holecek, J. J. , Baber, M. A. , Goldberg, V. M. , & Caplan, A. I. (1990). Bone and cartilage formation in diffusion chambers by subcultured cells derived from the periosteum. Bone, 11(3), 181–188. 10.1016/8756-3282(90)90212-H 2390376

[cre2287-bib-0026] Pinholt, E. M. , Ruyter, I. E. , Haanaes, H. R. , & Bang, G. (1992). Chemical, physical, and histologic studies on four commercial apatites used for alveolar ridge augmentation. Journal of Oral and Maxillofacial Surgery, 50(8), 859–867 discussion 867‐868. 10.1016/0278-2391(92)90280-D 1321896

[cre2287-bib-0027] Sasano, Y. , Kamakura, S. , Homma, H. , Suzuki, O. , Mizoguchi, I. , & Kagayama, M. (1999). Implanted octacalcium phosphate (OCP) stimulates osteogenesis by osteoblastic cells and/or committed osteoprogenitors in rat calvarial periosteum. The Anatomical Record, 256(1), 1–6. 10.1002/(SICI)1097-0185(19990901)256:1<1::AID-AR1>3.0.CO;2-X 10456979

[cre2287-bib-0028] Sibai, T. , Morgan, E. F. , & Einhorn, T. A. (2011). Anabolic agents and bone quality. Clinical Orthopaedics and Related Research, 469(8), 2215–2224. 10.1007/s11999-010-1722-9 21132409 PMC3126945

[cre2287-bib-0029] Suzuki, Y. , Kamakura, S. , Honda, Y. , Anada, T. , Hatori, K. , Sasaki, K. , & Suzuki, O. (2009). Appositional bone formation by OCP‐collagen composite. Journal of Dental Research, 88(12), 1107–1112. 10.1177/0022034509351378 19897786

[cre2287-bib-0030] Tallgren, A. (2003). The continuing reduction of the residual alveolar ridges in complete denture wearers: A mixed‐longitudinal study covering 25 years. The Journal of Prosthetic Dentistry, 89(5), 427–435. 10.1016/S0022-3913(03)00158-6 12806317

[cre2287-bib-0031] Tanuma, Y. , Matsui, K. , Kawai, T. , Matsui, A. , Suzuki, O. , Kamakura, S. , & Echigo, S. (2013). Comparison of bone regeneration between octacalcium phosphate/collagen composite and beta‐tricalcium phosphate in canine calvarial defect. Oral Surgery, Oral Medicine, Oral Pathology, Oral Radiology, 115(1), 9–17. 10.1016/j.oooo.2011.12.029 22901651

[cre2287-bib-0032] Zerbo, I. R. , Zijderveld, S. A. , de Boer, A. , Bronckers, A. L. , de Lange, G. , ten Bruggenkate, C. M. , & Burger, E. H. (2004). Histomorphometry of human sinus floor augmentation using a porous beta‐tricalcium phosphate: A prospective study. Clinical Oral Implants Research, 15(6), 724–732. 10.1111/j.1600-0501.2004.01055.x 15533134

